# Role of mitochondrial metabolism in immune checkpoint inhibitors-related myocarditis

**DOI:** 10.3389/fcvm.2023.1112222

**Published:** 2023-01-24

**Authors:** Xin Zhang, Yi Gan, Haoshuai Zhu, Zhihao Liu, Xiaojing Yao, Chao Cheng, Zhenguo Liu, Chunhua Su, Jianyong Zou

**Affiliations:** Department of Thoracic Surgery, The First Affiliated Hospital of Sun Yat-sen University, Guangzhou, China

**Keywords:** immunity therapy, myocarditis, immune checkpoint inhibitors, proteomics, mitochondrial metabolism

## Abstract

**Background:**

Immune checkpoint inhibitor-related myocarditis is the deadliest complication of immunotherapy. However, the underlying pathophysiological mechanisms of its occurrence and development remain unclear. Due to the long-term lack of effective early diagnosis and treatment options, it is of great significance to understand the pathophysiological mechanism of immune checkpoint inhibitor-related myocarditis.

**Methods:**

Tissue samples from three patients with immune checkpoint inhibitor-related myocarditis and three control tissue samples were collected for protein analysis. Differentially expressed proteins were screened out using quantitative proteomics technology based on TMT markers. Protein–protein interaction (PPI) and Gene Ontology (GO) functional enrichment analyses of cross-factors were subsequently performed. Combined with the PD-L1 subcellular organelle- level protein interaction network, we searched for hub proteins involved in immune checkpoint inhibitor-related myocarditis and explored potential drug sensitivity and disease correlation.

**Results:**

A total of 306 differentially expressed proteins were identified in immune checkpoint inhibitor-related myocarditis. Enrichment analysis showed that the differentially expressed proteins were closely related to mitochondrial metabolism. By analyzing mitochondria-related proteins and PD-L1-related proteins, we found four hub proteins, mammalian target of rapamycin (mTOR), Glycogen synthase kinase 3β (GSK3β), Protein tyrosine phosphatase non-receptor type 11 (PTPN11), and Mitofusin 2 (MFN2), indicating that they are closely related to immune checkpoint inhibitor-related myocarditis. Finally, we explored potential drugs for the treatment of immune checkpoint inhibitor-related myocarditis.

**Conclusion:**

Mitochondrial metabolism is involved in the process of immune checkpoint inhibitor-related myocarditis, and we identified four hub proteins, which may become new biomarkers for the early diagnosis and treatment of immune checkpoint inhibitor-related myocarditis.

## Background

Immune checkpoint inhibitors (ICIs), including PD-L1/PD-1 inhibitors, have recently emerged as potent therapies for a wide range of cancers, many of which are first-line treatments and can even improve outcomes for malignancies considered incurable (1). Accumulating evidence shows that cancer cells can exhibit high PD-L1 expression (2–4). The binding of PD-L1 to PD-1 on CD8+ T cells leads to the inactivation of effector functions of T-cells and the inhibition of T cell proliferation, thereby endowing cancer cells with strong immune evasion properties. Blocking the PD-L1/PD-1 axis restores T-cell function and induces durable tumor remission in cancer patients (4).

While enhancing antitumor immunity, ICIs also have autoimmune effects on normal tissues, and such adverse events, including myocarditis, are called immune-related adverse events (irAEs) (5, 6). A retrospective analysis reported that myocarditis occurred in 0.41% of patients receiving ICIs alone and 1.33% of patients receiving ICIs in combination, and mortality in patients with myocarditis is approximately 50%, making it the most lethal complication (7, 8). However, early detection of ICI-related myocarditis has become a challenge because the current mechanism of ICI-related myocarditis is unknown, and the diagnosis of this heart disease is very difficult until symptoms occur. Because PD-L1/PD-1 inhibitors are widely used; Thus, it is of great clinical and scientific significance to explore the mechanism of PD-L1/PD-1 in ICI-related myocarditis and strategies to reduce adverse reactions.

In recent years, high-throughput proteomics technology developed rapidly and became one new direction for exploring biological mechanisms. Defining biological processes based solely on gene expression without understanding the downstream changes in proteome is insufficient, as proteins are key functional drivers of biology and drug therapy targets (9, 10). Therefore, a more systematic study of the proteomic alterations in ICI-related myocarditis might shed light on the pathogenesis of this disease.

In the present study, we obtained endocardium tissues from patients with ICI-related myocarditis, compared proteomic profiles with viral myocarditis endocardium to identify differentially expressed proteins (DEPs) and analyzed their functions. We then explored the underlying mechanisms affecting ICI-related myocarditis and constructed potential pharmacology and disease networks. This study provides a comprehensive understanding of protein expression profiles involved in myocarditis and elucidates the underlying mechanisms in ICI-related myocarditis.

## Materials and methods

### Sample collection

All specimens were obtained from the First Affiliated Hospital of Sun Yat-sen University. Samples from patients with ICI-related myocarditis were used as the experimental group, patients were diagnosed with esophageal cancer and diagnosed with ICI-associated myocarditis after receiving immunotherapy alone. The main symptom of ICI-related myocarditis is shortness of breath, other symptoms may include fatigue, chest pain, and myalgia. The initial examination of suspected ICI-related myocarditis mainly includes ECG, troponin, NT-proBNP, two-dimensional transthoracic echocardiography and cardiac magnetic resonance. Endomyocardial biopsy (EMB) is recommended in cases where immune myocarditis is highly suspected by cardiologists and oncologists (11). All patients in this study underwent intramyocardial biopsy (EMB), and ICI-associated myocarditis was evaluated by experienced pathologists, mainly manifested by lymphocyte (CD4+, CD8+) and macrophage infiltration and interstitial fibrosis observed under the microscope (12). The control group was samples from patients with viral myocarditis. Specimens were collected 30 min after biopsy, immediately transferred to sterilized vials, frozen in liquid nitrogen, and stored at −80°C. The Clinical Research and Animal Trials Ethics Committee of the First Affiliated Hospital of Sun Yat-sen University approved this study.

### Sample processing

All samples were removed in a liquid nitrogen environment and then ground into powder. Sample powders were homogenized in lysis buffer (2.5% SDS/100 mM Tris–Cl, pH 8.0), sonicated for 10 min in an ice-water bath, and centrifuged at 12,000 × *g* for 15 min, and the supernatant was collected. The protein in solution was precipitated by acetone precipitation. Reconstitution solution (8 M urea/100 mM Tris–Cl, pH 8.0) was added to the obtained protein precipitate to redissolve it, dithiothreitol (DTT) was added to a final concentration of 10 mM, and the mixture was incubated at 37°C for 1 hour to allow the reduction reaction to proceed to open the disulfide bond. Then, iodoacetamide (IAA) was added to a final concentration of 40 mM, and the alkylation reaction was carried out at room temperature in the dark to block the thiol group. Protein concentration was determined by the Bradford method. Fifty micrograms of quantified protein samples were taken for SDS–PAGE detection, and protein bands were observed by Coomassie brilliant blue staining. First, 100 mM Tris–HCl solution (pH 8.0) was added to the protein sample. Then, the concentration of urea was diluted to below 2 M, and trypsin was added to obtain an enzyme protein mass ratio of 1:50. The mixture was incubated at 37°C with shaking overnight to carry out enzyme digestion. The next day, TFA was added to stop the enzyme digestion, and the pH value of the solution was adjusted to approximately 6.0, the solution was centrifuged at 12,000 × *g* for 15 min, and the supernatant was taken for desalting with Sep-Pak C18. The desalted peptide solution was dried using by a centrifugal concentrator, and then frozen at −20°C for later use.

An equal amount of sample was taken for TMT labeling. The labeling process was performed according to the instructions of the TMT manufacturer (Thermoscientific, Waltham, MA, USA). After the labeled samples were mixed in equal amounts, Sep-Pak C18 was used for desalination. After vacuum drying, the mixed samples were fractionated by high pH reverse chromatography, and finally combined into 15 fractions. After being vacuum-dried, the samples were stored in a −80°C refrigerator and was ready for testing on the machine.

Mass spectrometry was performed using a Q Exactive HF-X LC/MS system from Thermo Company. Peptide samples were aspirated by an autosampler, bound to a C18 trap column, and then eluted through to an analytical column (75 μm × 250 mm, 3 μm particle size, 100 Å pore size, Acclaim PepMap C18 column, Thermo) for separation. Two mobile phases (mobile phase A: 0.1% formic acid and mobile phase B: 80% ACN, 0.1% formic acid) were used to establish the analytical gradient. The liquid phase flow rate was modified to 300 nL/min. For mass spectrometry in DDA mode, each scan session consisted of a full MS scan (*R* = 120 K, AGC = 3e6, max IT = 50 ms, scan range = 350–1,800 m/z), followed by a 20 MS/MS scan (*R* = 60 K, AGC = 2e5, max IT = 110 ms). The DCD collision energy was set to 32. The screening window for the quadrupole was adjusted to 1.2 Da. The dynamic exclusion time was set to 40 s for the repeated acquisition of ions.

### Proteomics data analysis

MaxQuant (V1.6.6) software was used to retrieve mass spectrometry data, and the retrieval method was Andromeda. The SwissProt proteome reference database for humans in UniProt was used for searching. The main search parameters were as follows: TMT for item type; Oxidation (M), Acetyl (Protein N-term) for variable modification; Carbamidomethyl (C) for fixed modification; and enzymatic Trypsin/P for enzyme digestion. The search results were filtered according to 1% FDR at the protein and peptide levels, the anti-storage proteins, contaminating proteins, and protein entries with only one modified peptide were removed, and the rest of the peptide information was used for further analysis.

### Differential protein screening

The detection signal intensity of each peptide can be obtained by searching the database of raw mass spectrometry data, and then the quantitative information of the corresponding protein can be calculated. After normalization of the results, quantitative comparisons of the same protein between different samples were performed. According to the grouping of samples, after effective data screening and missing data filling, the protein quantitative ratio distribution in samples of each comparison group was calculated according to the protein quantitative data. A comparison ratio ratio >1.2 or <1/1.2 (*P* < 0.05) was used as the screening standard for differential proteins. Heatmaps were generated by the ggplot2 package of R language.^1^

### GO and KEGG pathway enrichment analyses

Gene Ontology (GO) enrichment analysis of these clusters was performed to explore their potential biological functions through ToppGene Suite.^2^ By integrating the kyoto encyclopaedia of genes and genomes (KEGG),^3^ the gene list of hub pathways was collected (12). The GO analysis mainly includes three aspects: cell composition analysis (CC), biological process analysis (BP), and molecular function analysis (MF). *p*-value <0.05 were considered significant for annotation analyses.

### Integration of the PPI-network

Protein–protein interactions (PPIs) were studied by STRING software ver. 11.0.^4^ The STRING database (version 10.1) was used to predict PPIs, screen closely related proteins and exclude irrelevant proteins. The confidence scores for linkage evaluation of PPIs were calculated, and 0.7 was set as the optimal cutoff value for PPIs. In addition, the hub module was identified using the Cytoscape plugin MCODE.

### PDL1 protein localization and interaction

ComPPI^5^ is an open, innovative, multidimensional PPI database that integrates information from multiple databases and provides useful protein information, such as interactions and their positioning (13). We obtained the PPI information of PDL1 from the ComPPI website, and the “ID mapping” function of the UniProt database was used to annotate the protein information in the PPI network.

### Drug sensitivity and disease correlation analysis of the hub genes

The altered gene sets and disease expression profiles of different drug treatments were retrieved from the BIOCARTA pathway data in the MSigDB database^6^ (14), and the potential drug targets of hub genes and disease expression analysis were analyzed by GSEA. A *p*-value <0.05 was considered significant.

## Results

### Differentially expressed protein screening

**FIGURE 1:**
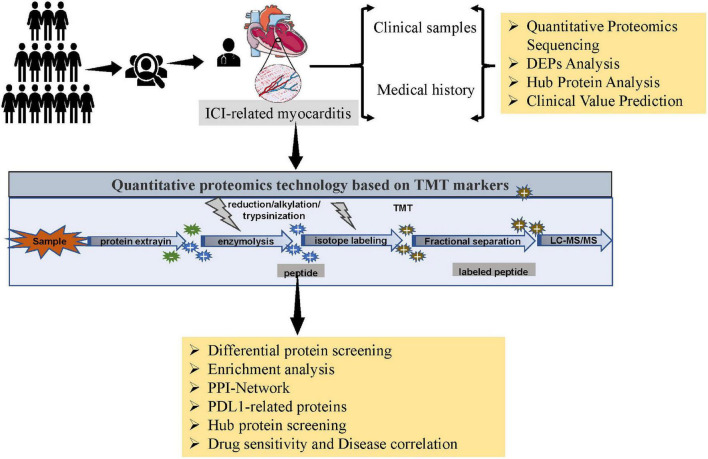
Flowchart.

**FIGURE 2:**
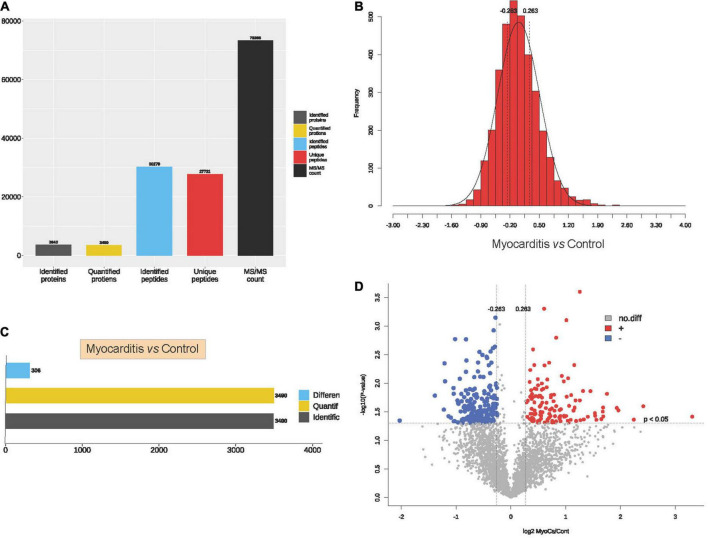
Identification of proteins in immune checkpoint inhibitors (ICI)-related myocarditis. **(A)** Overview diagram of protein identification. **(B)** Quantitative ratio distribution map of immune myocarditis and normal myocardial tissue. **(C)** Differential protein screening between immune myocarditis and normal myocardial tissue. **(D)** Volcano map of differential protein screening.

To elucidate the proteomic features of ICI- related myocarditis, 3 ICI-related myocarditis tissues and 3 viral myocarditis tissues were collected and further sequencing using TMT-labeled LC–MS/MS. The research process is shown in the flowchart (Figure 1). Through mass spectrometry, 3,640 proteins were found, 3,490 of which had quantitative information (Figure 2A). After normalizing the raw data of mass spectrometry, the quantitative ratio distribution of proteins in ICI-related myocarditis and viral myocarditis groups can be calculated according to the quantitative protein data, and the results are shown in Figure 2B. With a ratio >1.2 or <1/1.2 and *P* < 0.05 as the screening criteria for DEPs, 306 DEPs were found in ICI-related myocarditis and viral myocarditis tissue, of which 124 proteins were upregulated proteins and 182 proteins were downregulated proteins (Figures 2C, D).

### Protein enrichment analysis and protein–protein interactions

**FIGURE 3:**
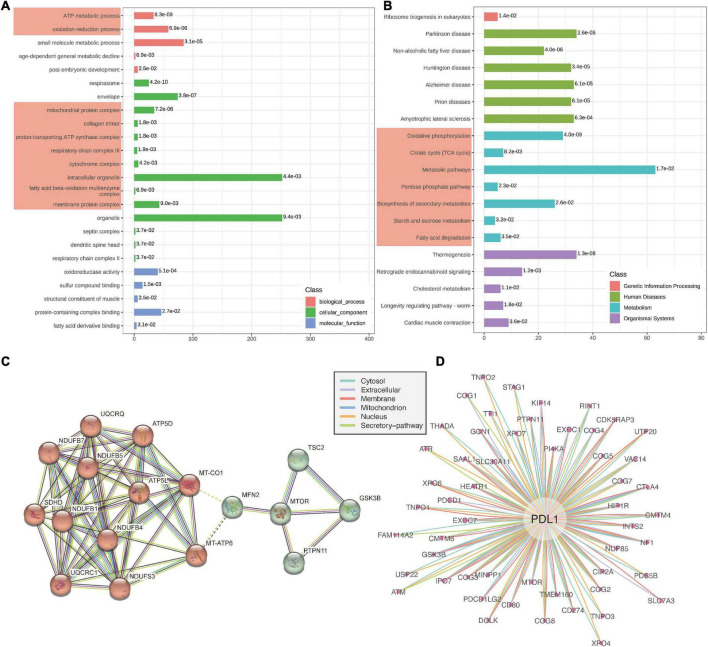
Protein enrichment analysis and protein–protein interactions (PPIs). **(A)** Gene Ontology (GO) enrichment analysis of differentially expressed proteins. **(B)** Kyoto encyclopaedia of genes and genomes (KEGG) enrichment analysis of differentially expressed proteins. **(C)** PPI network. **(D)** PD-L1 subcellular organelle protein interaction network.

Through GO enrichment analysis of the differentially expressed proteins, we found that they were mainly involved in the BPs of ATP energy metabolism, oxidation-reduction reduction and mitochondrial protein complex, the CCs of mitochondrial protein complex and collagen trimer, and the MFs of oxidoreductase activity and dendritic spine head (Figure 3A). KEGG enrichment analysis showed that the differentially expressed proteins mainly involved in the signaling pathways of oxidative phosphorylation, citrate cycle, and metabolic pathways (Figure 3B). By constructing a PPI network, we identified 17 core proteins (Figure 3C). Here, combining the above differential pathway analysis results, we found that mitochondrial energy metabolism may play important regulatory roles in the occurrence and progress of ICI-related myocarditis.

### Hub proteins between mitochondrial metabolism and ICI-related myocarditis

**FIGURE 4:**
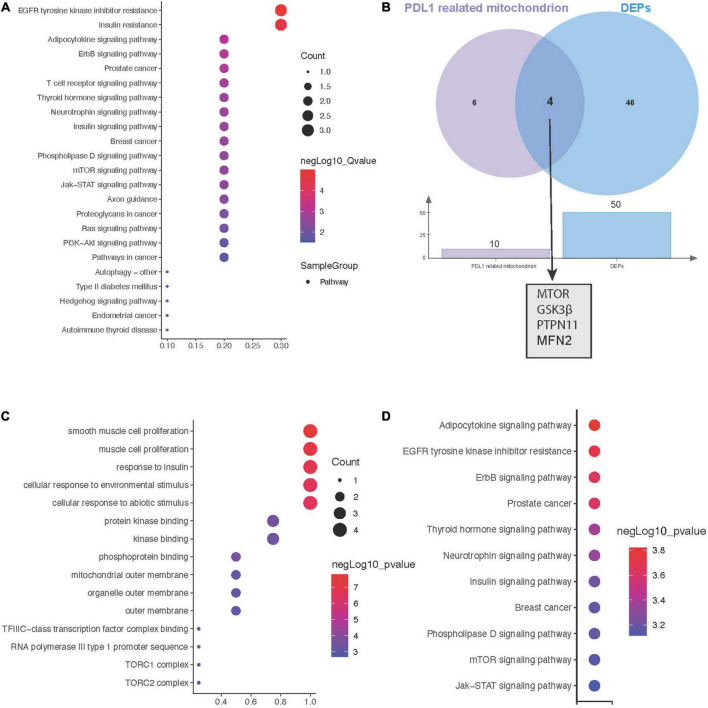
Hub genes between mitochondrial metabolism and immune checkpoint inhibitors (ICI)-related myocarditis. **(A)** Kyoto encyclopaedia of genes and genomes (KEGG) enrichment analysis of PD-L1-related proteins. **(B)** Screening for coexpressed proteins between PDL1 and mitochondria-related proteins in differentially expressed proteins (DEPS). **(C)** Biological process (BP) enrichment analysis of hub proteins. **(D)** KEGG enrichment analysis of hub proteins.

Due to the key role of PD-L1 in immunotherapy, we further constructed a protein interaction network at the subcellular organelle level (Figure 3D). Enrichment analysis by KEGG, revealed that PDL1-related proteins are mainly involved in the regulation of epidermal growth factor receptor (EGFR) tyrosine kinase inhibitor resistance, insulin resistance, the Erbb signaling pathway and prostate cancer (Figure 4A). Then, we analyzed the coexpressed proteins between mitochondria-related proteins in the PDL1-related proteins and DEPs, and obtained four hub proteins, TOR, Glycogen synthase kinase 3β (GSK3β), Protein tyrosine phosphatase non-receptor type 11 (PTPN11), and Mitofusin 2 (MFN2) (Figure 4B). GO analysis showed that the hub proteins were mainly involved in biological processes, such as smooth muscle cell proliferation, muscle cell proliferation and response to insulin (Figure 4C). KEGG analysis showed that the hub proteins were mainly involved in the regulation of pathways, such as adipocytokine signaling, EGFR tyrosine kinase inhibitor resistance, and the Erbb signaling pathway (Figure 4D).

### Drug sensitivity and disease correlation analysis of hub proteins

**FIGURE 5:**
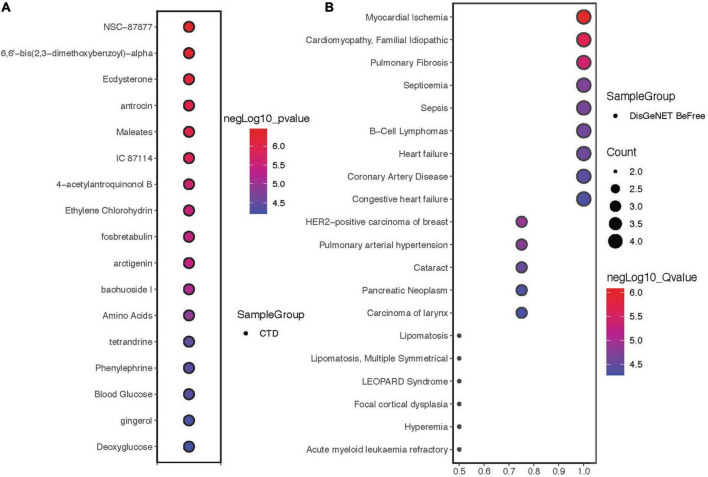
Drug sensitivity and disease correlation analysis of hub proteins. **(A)** Drug sensitivity correlation analysis of hub proteins. **(B)** Disease correlation analysis of hub proteins.

Drug sensitivity showed that the hub proteins were mainly sensitive to NSC-87877, 6,6′-bis(2,3-dimethoxybenzoyl)-α, ecdysterone and other drugs (Figure 5A). The 4 proteins are mainly expressed in myocardial ischemia, cardiomyopathy, familial idiopathic pulmonary fibrosis and other diseases (Figure 5B).

## Discussion

In recent years, immune checkpoint inhibitors have been widely used in clinical practice to improve the prognosis of patients with malignant tumors. Myocarditis caused by ICIs has also received attention. Although its incidence is not that high, it is usually severe and life-threatening (15). In this study, we aimed to determine the potential factors involved in ICI-related myocarditis through proteomic analysis and provide a theoretical basis for the early detection and treatment of immune-related myocarditis.

By comparing protein differences between ICI-related myocarditis and control samples, we found that mitochondrial metabolism may be the significant metabolic change in immune-related myocarditis. Mitochondria provide most of the biological energy for biological processes. As one of the most important organelles, they are the most important site for catabolism and are also a key regulator of cell proliferation and apoptosis (16). Mitochondrial function is critical in cardiomyocytes because they not only continuously produce ATP to maintain normal cardiac function, but also play an important role in apoptosis, calcium homeostasis (17). Mitochondria are also an important place to generate Reactive oxygen species (ROS). ROS play an important role in the immune system by increasing the phagocytosis of intracellular pathogens and transmitting danger signals. In addition, ROS are involved in cell signaling, proliferation and apoptosis. However, overproduction of ROS can lead to damage to host cells, leading to various diseases, including respiratory, cardiovascular, and digestive disorders (18). The high density of mitochondria in cardiomyocytes and the dependence of cardiomyocytes on mitochondria make immune-related myocarditis closely related to changes in mitochondrial function (19). Abnormal mitochondrial energy metabolism pathways are also associated with oxidative stress, which can cause DNA damage, impairment of homeostasis, and tumorigenicity (20). Mitochondrial dynamics influence immune cell activation, differentiation and cytokine production. As the main organelle for energy supply, mitochondria provide ATP to support various physiological activities of T cells. Furthermore, the direction of T cell migration is regulated subcellularly by mitochondria. Mitochondria cluster near the cell edge by supplying extracellular chemokines with sufficient ATP for T cell recruitment to tumor sites (21).

PD-1/PD-L1 inhibitors take effect by preventing binding of PD-L1/PD-L1 and restoring the function of immune T cells (22). ICIs can also lead to heart damage, and some studies have found that the injured myocardial tissue has a large number of infiltrated lymphocytes, such as T lymphocytes and macrophages (23). PD-L1 expression is not only expressed on immune cells, but also in self-tissues and several tumor cell, which induces immunosuppression (24). There are two main aspects of how the immune system damages the heart: (a) ICIs interfere with PD-L1/PD-L1 signaling in the heart by lowering the threshold of T cell activation, which results in the destruction of peripheral immune tolerance. (b) ICIs may target T-cells that share epitopes with the tumor and myocardium, and ICIs can enhance T-cell effector function and lead to the development of ICI-related myocarditis (25). Combining the above analyses, we found that PD-L1 and mitochondrial metabolism may be involved together in the progress of ICI-related myocarditis. Mitochondria are closely related to the immune microenvironment, which has been confirmed in some studies. Firstly, T cell activation is inseparable from mitochondria, as ROS production is required for nuclear factor of activated T cells (NFAT) to activate and induce inflammatory cytokines, whereas ROS precursors can block PD-1 by expanding effector/memory T cells in lymph nodes and tumors, thereby synergistically enhancing tumoricidal activity (26, 27). Additionally, mitochondria involve in T cell exhaustion. Mitochondrial respiration maintains cellular homeostasis by regulating endolysosomal function and also regulates T cell function, and loss of mitochondrial function leads to dysfunctional T cell differentiation (28). By analyzing coexpressed proteins, we found four hub proteins that may lead to the occurrence of ICI-related myocarditis, mammalian target of rapamycin (mTOR), GSK3β, PTPN11, and MFN2.

Mammalian target of rapamycin regulates cellular processes and biological homeostasis by participating in mitochondrial function, fatty acid metabolism, glycolysis, the oxidative stress response, and other metabolic pathways (29). Studies have found that mTOR regulates protein synthesis through phosphorylation to maintain cardiac structure and function (30). In addition, mTOR is involved in the recruitment of immune cells to the heart, which in turn leads to cardiac remodeling (31). GSK3β is a serine/threonine protein kinase signaling molecule widely expressed in a variety of cell types. GSK3β phosphorylation affects glycogen metabolism and is involved downstream of insulin signaling (32). The NLRP3 inflammasome is reported to be one of the key factors leading to myocarditis. GSK-3β inhibitors can antagonize the effect of the NLRP3 inflammasome by reducing the proliferation of monocytes and macrophages, which alleviates the immune response (33, 34). MFN2 encodes a GTPase protein located on the outer mitochondrial membrane and was originally found in smooth muscle cells (35). As a mitochondrial fusion protein, MFN2 participates in the process of heart disease by regulating proteolysis and inducing mitochondrial oxidative stress (36). PTPN11 is known to be a signaling molecule that regulates a variety of cellular processes, including cell growth, differentiation, the mitotic cycle, and oncogenic transformation (37). It is widely involved in body activities, such as cell cycle progression and cell proliferation by regulating phosphatases and changing protein activity (38). PTPN11 is involved in tumorigenesis by inducing excessive activation of mitochondrial function; however, its role in myocarditis remains unclear (39).

We subsequently investigated the drug sensitivity and disease associations of hub genes and found that NSC-87877, bis(2,3-dimethoxybenzoyl)-α and ecdysterone may be effective for the treatment of ICI-related myocarditis. NSC-87877 is an SHP-2 inhibitor that inhibits phosphorylation and prevents the accumulation of inflammatory cells (40). Bis(2,3-dimethoxybenzoyl)-α is a trehalose derivative from brartemicin that treats disease by repairing oxidative stress damage (41, 42). Ecdysterone, a naturally occurring ecdysone, can significantly reduce ROS production and reduce mitochondrial membrane depolarization (43). Finally, we investigated the correlation between these four proteins and diseases and found that they were closely related to heart diseases, which was consistent with our expectations.

To our knowledge, this study is the first to reveal mitochondrial mechanism of ICI-related myocarditis by proteomics. Our findings provide new insights into the pathogenesis and treatment of ICI-related myocarditis, and provide a theoretical basis for early identification of ICI-associated myocarditis.

This study has some limitations. First, our study was limited by the relatively small sample size in the proteomics analysis, future studies with larger sample sizes are needed to confirm these findings. Second, we confirmed that mTOR, GSK3β, PTPN11 and MFN2 are highly expressed in ICI-related myocarditis by proteomic sequencing, cellular experiments are required to verify our hypothesis. In addition, the partial outcomes obtained by signaling pathways and drugs related to ICI-related myocarditis, which provided predictive value for future clinical work, need further clinical verification and experimental research. Finally, to reduce the number of false positive DEPs, we obtained co-expressed DEPs in the experimental group samples. In this way, many important genes may have been overlooked. Despite the limitations, this study provides a new perspective that mitochondrial metabolism involves in the process of ICI-associated myocarditis, which might become the foundation of diagnosis and treatment of ICI-associated myocarditis.

## Conclusion

In this study, we found the protein profile of ICI-associated myocarditis by quantitative proteomics based on TMT markers. Through functional analysis of the 306 identified DEPs, we identified the involvement of mitochondrial metabolism in pathogenic progress. By combining PD-L1-related proteins, we found that mTOR, GSK3β, PTPN11, and MFN2 may be potential diagnosis and treatment biomarkers of ICI-associated myocarditis.

## Data availability statement

All of the raw data has been deposited to the ProteomeXchange Consortium *via* the iProX partner repository with the dataset identifier IPX0005756000.

## Ethics statement

The studies involving human participants were reviewed and approved by the Clinical Research and Animal Trials Ethics Committee of the First Affiliated Hospital of Sun Yat-sen University. Written informed consent for participation was not required for this study in accordance with the national legislation and the institutional requirements.

## Author contributions

JZ designed the study. XZ, YG, and HZ performed the analysis and wrote the manuscript. ZhiL, ZheL, and XY contributed to preparing the figures. JZ, CS, and CC revised the manuscript. All authors reviewed the manuscript and approved the final version.

## Footnotes

1
https://cloud.r-project.org/package=ggplot2

2
https://toppgene.cchmc.org

3
http://www.genome.ad.jp/kegg/

4
https://string-db.org/

5
https://comppi.linkgroup.hu/

6
http://www.gsea-msigdb.org/gsea/msigdb/index.jsp
